# Construction of viral-based expression vectors for high-level production of human interferon alpha 2b in plants

**DOI:** 10.1007/s00253-024-13069-7

**Published:** 2024-02-23

**Authors:** Yana Sindarovska, Mykola Kuchuk

**Affiliations:** Department of Genetic Engineering, Institute of Cell Biology and Genetic Engineering of NAS of Ukraine, Akad. Zabolotnogo Str., 148, Kyiv, 03148 Ukraine

**Keywords:** Human interferon alpha 2b, Transient gene expression, *Nicotiana benthamiana*, *Ocimum basilicum*, Recombinant proteins, Potato virus X (PVX)-based vector

## Abstract

**Abstract:**

Human interferon (hINF) alpha 2b is clinically important pharmaceutical product included in combinatory therapy against chronic hepatitis C and B and complex therapy against several cancer diseases. Here, we created the genetic constructions, based on genome elements of potato virus X (PVX), carrying the *infα2b* gene for transient expression in plant cells. The created plasmid vector constructions were tested through *Agrobacterium*-mediated transient gene expression method in two plant species—*Nicotiana benthamiana* and *Ocimum basilicum* (sweet basil). Production of recombinant hINF alpha 2b was more efficient in *N. benthamiana* than that in *O. basilicum* plants. The average yield of hINF alpha 2b produced in *N. benthamiana* plants was 0.56 mg/g of fresh leaf weight (FW) or 6% of the total soluble cell proteins (TSP). The maximal level reached up to 1.2 mg/g FW or 9% TSP. We estimated that about 0.67 mg of hINF can be obtained from one *N. benthamiana* plant. The yield of hINF alpha 2b obtained with the PVX-based expression cassette was about 80 times higher than the yield of hINF alpha 2b obtained with a simple expression cassette in which the *infα2b* gene was controlled by the 35S promoter of cauliflower mosaic virus.

**Key points:**

***•*** *PVX-based expression vectors provide efficient transient expression of infα2b gene*

***•*** *N. benthamiana plants can produce human interferon alpha 2b at high levels*

***•*** *The yield of the hINF α2b reached up to 1.2 mg/g of fresh leaf weight*

## Introduction

Interferons are multifunctional secretory glycoproteins, and cytokines, produced by host immune cells in response to pathogen attack. Nowadays, the global interferons market is valued at USD 8.5 billion (Interferons Global Market Report 2023). Human leukocyte interferon (hINF) alpha 2b is one of the important biopharmaceuticals, as it is included in antiviral therapy of chronic hepatitis C and B (Gull et al. 2019; Ye and Chen 2021) and other viruses, including recently emerged pandemic virus SARS-CoV2 (Pandit et al. 2021; Xu et al. 2022), treatment of multiple sclerosis, as well as complex anticancer therapy against hairy cell leukemia, melanoma, follicular lymphoma, renal cell carcinoma, and several others (Ghaffari et al. 2021; Ningrum 2014). It has also been shown that hINF alpha 2b produced in plant systems reduced the level of the repair enzyme MGMT in Hep-2 cancer cells, thereby increasing the sensitivity of cancer cells to chemotherapy (Nidoieva et al. 2019).

While expression systems based on bacterial and mammalian cell cultures are dominant production of most recombinant proteins, including interferons (Castro et al. 2021), each of them has several limitations, e.g., existence of bacterial endotoxins, possible contamination with human pathogens, oncogenes or prions, and recombinant protein aggregation into insoluble inclusion bodies. These disadvantages can be overcome by alternative expression system, such as plant expression system. Plants have several advantages as producers of recombinant proteins: (i) ease of cultivation, scalability, and low costs, (ii) absence of endotoxins and human pathogens, (iii) formation of soluble fraction of target proteins, and (iv) post-translational modifications of eukaryotic proteins. The main limitation that restricts largely plant expression systems is the low final yield of recombinant proteins is especially noticable for stably transformed plant cells.

Transient gene expression is a rapid and simple approach that can significantly increase the production levels of target reporter recombinant proteins in plants up to 1–5 mg/g of fresh leaf weight (Marillonnet et al. 2005; Sindarovska and Kuchuk 2023; Yamamoto et al. 2018). The production levels of a recombinant protein in plant cells depend largely on (i) its own stability (Benchabane et al. 2008; Burnett and Burnett 2020), (ii) plant host species (Hoshikawa et al. 2019; Sheludko et al. 2007; Sindarovska et al. 2014, 2019; Torti et al. 2021; Wroblewski et al. 2005; Yamamoto et al. 2018), (iii) and the efficiency of expression system used (Eidenberger et al. 2023; Sheludko et al. 2007). While simple expression systems where the target gene is controlled by 35S promoter of cauliflower mosaic virus (CaMV) are often used for transient gene expression in plants (Norkunas et al. 2018; Pang et al. 2019; Phan et al. 2020), improved expression systems that include viral genome elements, such as genes coding viral polymerases, replication initiation protein, coat and movement proteins, and suppressors of gene silencing, can enhance the expression of the target genes, and increase the final yield of recombinant proteins (Jiang et al. 2019; Malla et al. 2021; Phakham et al. 2021; Poborilova et al. 2020; Sheludko et al. 2007; Sindarovska and Kuchuk 2023; Yamada et al. 2020; Yamamoto et al. 2018). Tobacco mosaic virus and potato virus X genomes are often used to create improved expression cassettes and to achieve high levels of a target protein through transient gene expression (Baró et al. 2022; Dickmeis et al. 2014; Lindbo 2007a, 2007b; Marillonnet et al. 2004; Shi et al. 2019; Sindarovska and Kuchuk 2021, 2023; Torti et al. 2021).

In this study, we created and tested the expression vectors based on genome elements of potato virus X (PVX) carrying the *inf α2b* gene for production of human interferon alpha 2b through *Agrobacterium*-mediated transient gene expression in plants.

## Materials and methods

### Initial genetic constructions

**Fig. 1:**
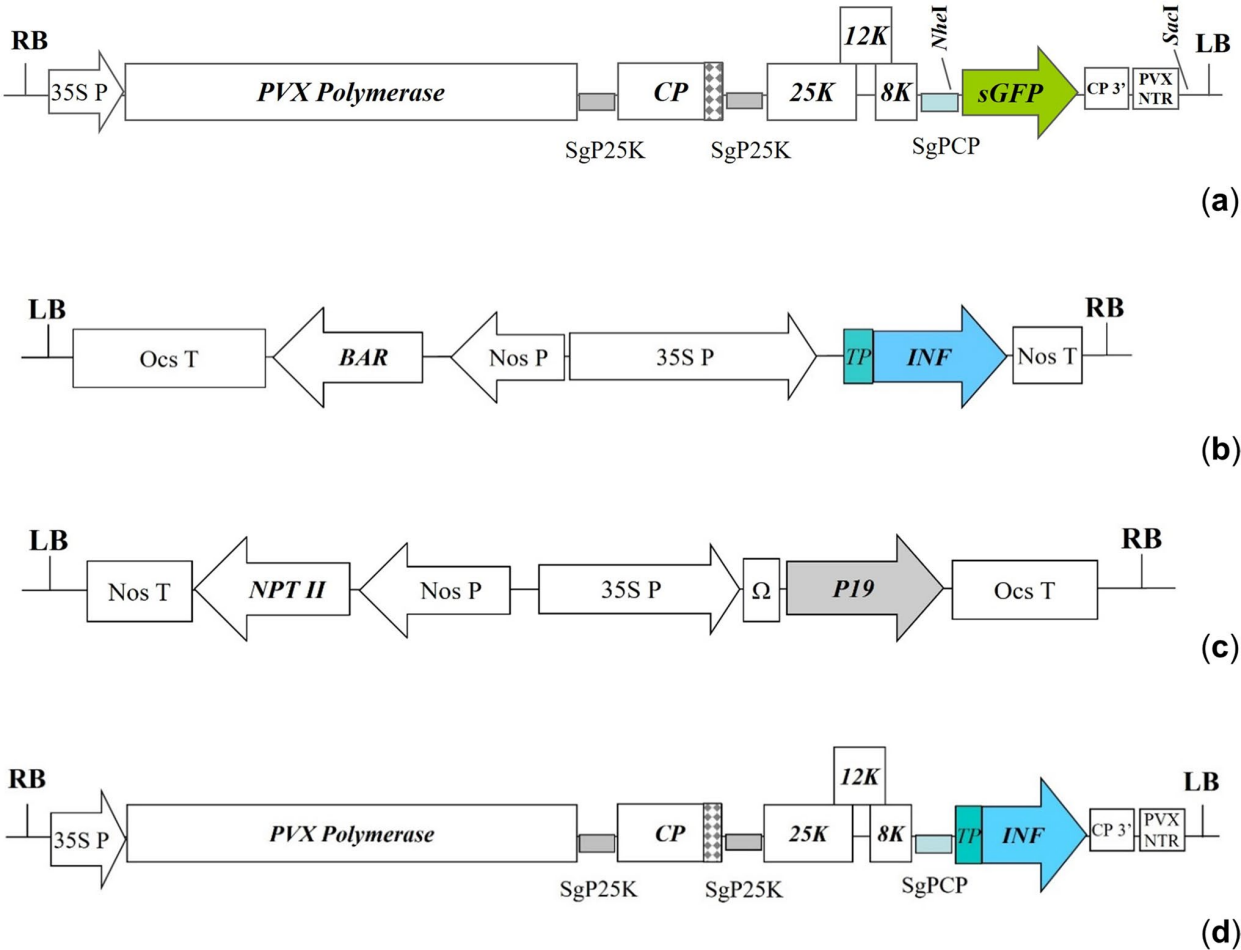
The graphic presentation of used and created expression cassettes: **a** pICH27566; **b** pCB125; **c** pICH6692; **d** pPVX-INF (a long vector version has 5′UTR before *transit peptide* sequence while a short version does not have): 35S P – 35S promoter of CaMV; SgP25K – subgenomic promoter of the PVX *25 K protein* gene; SgPCP – subgenomic promoter of the PVX *coat protein* gene; nos P, nos T – promoter and terminator of the *A. tumefaciens nopaline synthase* gene; ocs T – terminator of the *A. tumefaciens octopine synthase* gene; Ω – 5′ omega leader sequence of tobacco mosaic virus (translational enhancer); *sGFP* – the *gfp* gene coding reporter GFP; *INF –* the *inf α2b* gene coding human interferon alpha 2b fused in the open reading frame with *apoplast transit peptide* sequence (*TP*) (*calreticulin signal* from *N. plumbaginifolia*); *P19* – the gene coding P19 protein of tomato bushy stunt virus, suppressor of PTGS; *PVX Polymerase* – the gene coding PVX RNA-directed RNA polymerase; *CP* – the gene coding PVX coat protein; *25 K, 12 K, 8 K* – the triple gene block (the genes coding PVX 25 K, 12 K, and 8 K proteins); *BAR* – the gene coding bialaphos resistance (BAR) protein; *NPT II* – the gene coding neomycin phosphotransferase II; CP 3′ – 3′ end of the PVX *coat protein* gene; PVX NTR – 3′UTR of PVX; RB, LB – left and right borders of *A. tumefaciens* T-DNA

The pICH27566 plasmid vector contained all five open reading frames (ORFs) coding PVX proteins: RNA-directed RNA polymerase, coat protein, 25 K, 12 K, and 8 K proteins coded by the triple gene block; and the target *gfp* gene coding the green fluorescent protein (GFP) (Fig. 1a). The pICH27566 plasmid vector was generously donated by Nomad Bioscience GmbH Company (Halle, Germany) for scientific goals. The pCB125 plasmid vector carrying the target *inf α2b* gene (coding hINF alpha 2b) fused in ORF with the plant *apoplast transit peptide* (*TP*) sequence placed at the 5′-end and driven by 35S promoter of CaMV was obtained from the collection of the Institute of Cell Biology and Genetic Engineering, Kyiv, Ukraine (Fig. 1b, Gerasymenko et al. 2009). The pICH6692 plasmid vector carrying the target *p19* gene coding a viral suppressor of post-transcriptional gene silencing driven by 35S promoter of CaMV was generously donated by Nomad Bioscience GmbH Company (Halle, Germany) for scientific goals (Fig. 1c). The pICH6692 plasmid vector was used to enhance the target transgene expression during agroinfiltration procedure.

### Plant material and growth conditions

*Nicotiana benthamiana* seeds were obtained from the National Germplasm Bank of World Flora of the Institute of Cell Biology and Genetic Engineering (Kyiv, Ukraine). *Ocimum basilicum* seeds (green variety “Shyrokolystyy”) were bought in a local seed shop. Seeds were germinated in commercial soil and plants were grown in greenhouse conditions: 16-h light day at 24–26 °C, 3000–4000 lx. Six to 8-week-old plants were used for agroinfiltration experiments.

### Agroinfiltration procedure

Preparation of *Agrobacterium* suspension and agroinfiltration procedure were performed as described earlier (Sindarovska and Kuchuk 2021). Suspension of agrobacterial cells harboring the pPVX-INF plasmid vector (long or short version) (Fig. 1d) or pCB125 plasmid vector (Fig. 1b) was mixed in equal volume with suspension of agrobacterial cells harboring the pICH6692 plasmid vector (Fig. 1c) (pPVX-INF + pICH6692 or pCB125 + pICH6692). For the internal positive controls of good conditions for transient gene expression suspension of agrobacterial cells harboring the plasmid vectors with the *gfp* gene was used. The pICH27566 plasmid vector (Fig. 1a) was mixed in equal volume with suspension of agrobacterial cells harboring the pICH6692 plasmid vector (pICH27566 + pICH6692) to control conditions for gene expression in the PVX-based constructions. The pNMD2501 plasmid vector where the *gfp* gene was driven by 35S promoter CaMV (Sindarovska and Kuchuk 2023) was used to control conditions of gene expression in a simple vector system (pCB125). GFP can be easily detected in non-destructive conditions under long-wave UV light. Bright green fluorescence in leaves (corresponded to high GFP expression) confirmed good conditions for recombinant protein production. As the negative controls, we used intact leaves or leaves infected with suspension of agrobacterial cells harboring the pICH6692 plasmid vector only. The first to third upper leaves of *N. benthamiana* and the second to third upper leaves of *O. basilicum* plants were infiltrated with agrobacterial suspension using needleless syringe. Experiments were repeated twice and totally four to eight replications were done.

### Isolation of recombinant protein and protein yield analysis

The fresh leaf tissues were weighed and ground into fine powder at + 4 °C in a pre-cooled mortar with a pestle and then two volumes of ice-cold extraction buffer (w/v) were added (Sindarovska and Kuchuk 2021). To purify the protein fraction, two rounds of centrifugation were performed at 16,000 g for 10–15 min at + 4 °C. The Bradford method was used to calculate the content of total soluble proteins in the leaf extracts; the bovine serum albumin was used as a standard protein for calculation (Bradford 1976). Clarified protein extracts were tested for the presence of hINF alpha 2b using an enzyme-linked immunosorbent assay (ELISA). Indirect ELISA was carried out according to a protocol described by (https://www.usbio.net/protocols/elisa-methods). Just before coating the wells, the purified protein extracts were diluted in coating buffer (CB) (at least 1:20), and then serial dilutions were made in the 96-well plates. Protein extracts prepared from the leaves of uninfected plants, leaves infected with the pICH6692 plasmid vector, or leaves infected with the pICH27566 plasmid vector were used as negative controls. For the positive controls, we used standard hINF alpha 2b (product code ab51094, Abcam, UK) diluted in CB or standard hINF alpha 2b added to the protein extracts prepared from the leaves of uninfected plants. Primary antibodies (anti-interferon alpha 2b antibody [4E10], product code ab9388, or [9D3] product code ab9386, Abcam, UK) were diluted in a blocking buffer (1:1000) so the final antibody concentration in solution was 1 µg/ml. Secondary antibodies (goat anti-mouse IgG H&L (HRP), product code ab205719, Abcam, UK) were diluted in a blocking buffer (1:4000) so the final antibody concentration in solution was 0.5 µg/ml. After all required steps of incubation, final rinsing with a washing buffer, and desiccation, ultrasensitive 3,3′,5,5′-tetramethylbenzidine (TMB) solution (Cat. No. ES022-100 ml, Millipore, USA), a substrate for horseradish peroxidase, was applied. At the final step 0.3 Mol/L of sulfuric acid was added to each well to stop the reaction and results were read at 450-nm wavelength using a multi-mode microplate reader FLUOstar Omega (BMG LABTECH, Germany). The serial dilutions with known concentration of standard hINF alpha 2b diluted in CB were made for every set of experiments to make the calibration curve with a linear regression. To calculate the hINF in the experimental plant extracts, we chose the absorbance value for the experimental samples (minus the background absorbance value of the control extracts) that was within the linear regression interval. Then the value was multiplied by the dilution factor.

### Statistical data

Statistical data were calculated in the Excel 2016 program, Microsoft office software.

## Results

The pPVX-INF plasmid vectors were created based on the pICH27566 plasmid vector (an acceptor vector, Fig. 1a) by replacing the *gfp* gene with the target *inf α2b* gene. The native *inf α2b* gene fused with the plant apoplast *TP* sequence (calreticulin signal) was synthesized de novo using the pCB125 plasmid donor vector (Fig. 1b) as a template.

### Insertion preparation

To replace the *gfp* gene on the target *inf α2b* gene in the plasmid acceptor vector, two unique restriction sites (*Nhe*I and *Sac*I) closest to the gene of interest were chosen (Fig. 1a). *Nhe*I was located within subgenomic promoter of the *CP* gene (SgPCP), and *Sac*I was located after the 3′ non-translated region (NTR) of PVX. However, the *Sac*I site was located approximately 160 bp distal to the end of the *gfp* gene; so to obtain the same plasmid vector backbone, we prepared separately two fragments (one with the *inf α2b* gene and another with a part of NTR) which then were combined in one insertion fragment carrying the *Nhe*I and *Sac*I restriction sites at the 5′- and 3′-ends, respectively.

**Fig. 2:**
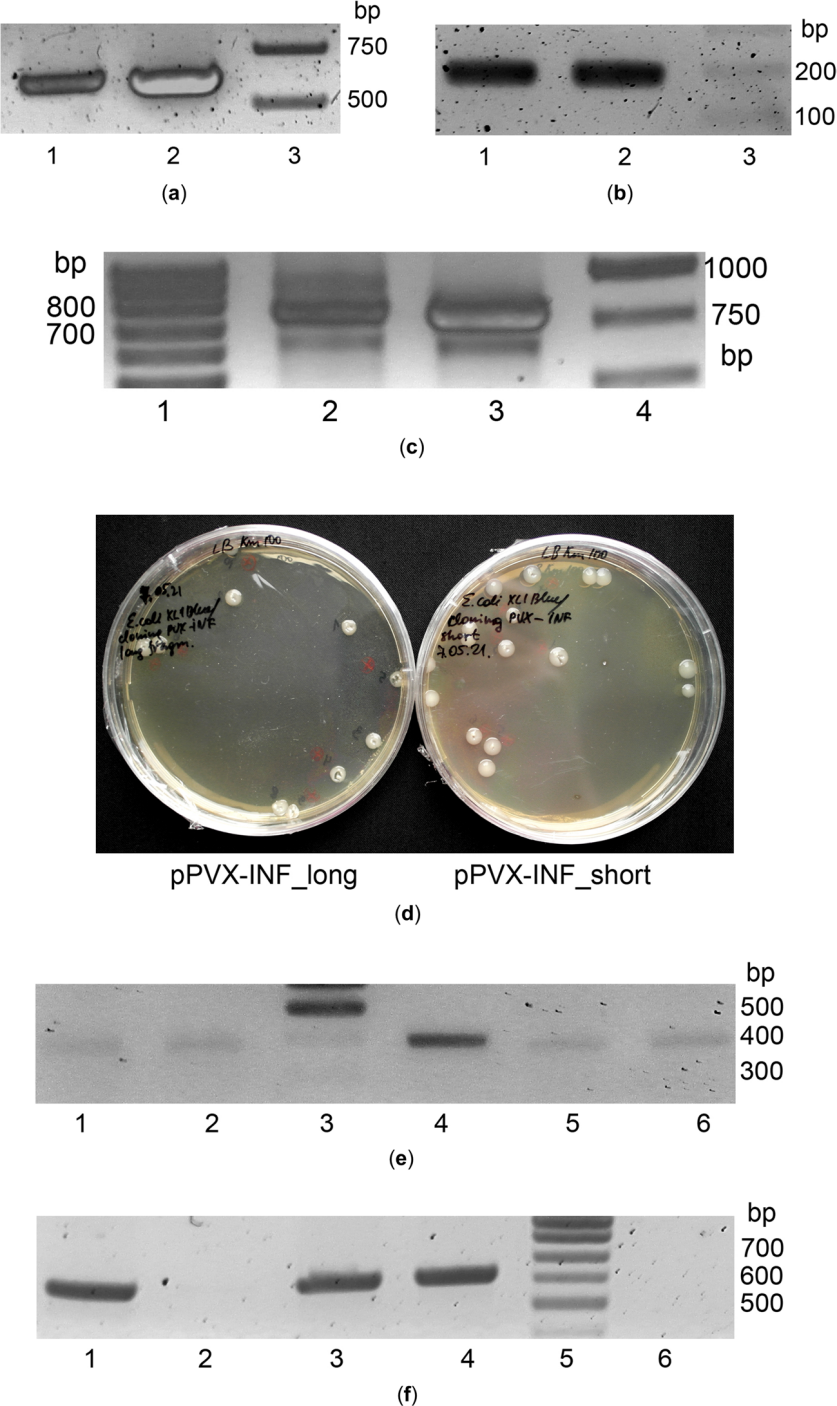
Creation and verification of the PVX-based plasmid vectors carrying the target *inf α2b* gene. **a–c** Preparation of insertions carrying the *inf α2b* gene fused with *TP* sequence with the 3′- and 5′-overhangs for cloning in the PVX-based vector (pICH27566). **a** PCR products of the *inf α2b* gene fused with *TP* sequence: lanes 1, 2 – the fragments A and B, correspondingly, lane 3 – DNA marker ladders, bp; **b** PCR products the part of NTR with *Sac*I restriction site: lanes 1, 2 – the fragments C, lane 3 – the DNA marker ladder, bp; **c** PCR products of the combined insertion fragments: lanes 1, 4 – DNA marker ladders, bp, lane 2 – the fragment D, lane 3 – the fragment F; **d** transformation of the pPVX-INF plasmid vectors (long and short versions) in *E. coli* competent cells: colonies carrying the pPVX-INF plasmid vectors were obtained on the LB medium with a selective agent; **e**,** f** Colony PCR analysis to verify the pPVX-INF plasmid vectors presence in the appeared *E. coli* colonies with two *inf α2b* gene specific pairs of primers: **e** lanes 1, 2, 5, 6 – the samples of *E. coli* colonies, 4 – a positive control: the plasmid vector pCB125 carrying the *inf α2b* gene, 3 – the DNA marker ladder, bp; **f** lanes 1–4 – the samples of *E. coli* colonies, 5 – the DNA marker ladder, bp; 6 – a negative control: the plasmid vector pCB125 (it can bind only a forward primer, but not a reverse one)

The *inf α2b* gene fused with *TP* sequence was amplified with a pair of primers YI-1 or YI-2 (forward) and YI-3 (reverse) (Table 1) using the pCB125 plasmid donor vector as a template. Two variants of the insert fragment were expected to be obtained after PCR amplification: one has 15 bp untranslated region (5′UTR) between a subgenomic promoter and a start codon (as in the acceptor vector)—a long insert variant; another fragment has a start codon located directly after promoter sequence—a short insert variant. Primers were designed in such manner that the resulting PCR products had the *inf α2b* gene fused with *TP* sequence and the 3′-and 5′-overhangs from the pICH27566 plasmid acceptor vector (a part of SgPCP with *Nhe*I restriction site at the 5′-end, and a part of NTR region at the 3′-end). Phusion High-Fidelity (HF) PCR Kit (Cat. No. F-553S, Thermo Fisher Scientific) was used for gene amplification. PCR was carried out according to the protocol recommended by the manufacturer. The PCR reaction mixture included 1x  Phusion HF Buffer, dNTPs mix (200 µM each), pair of primers (0.5 µM each), a template plasmid, Phusion HF DNA Polymerase (0.4 U), and milliQ water to the final volume 20 µL. The PCR amplification program was as follows: initial denaturation 98 °C – 30 s, 5 cycles (denaturation 98 °C – 10 s, primer annealing 52 °C – 15 s, extension 72 °C – 15 s), 30 cycles (denaturation 98 °C – 10 s, primer annealing 55 °C – 15 s, extension 72 °C – 15 s), final extension 72 °C – 7 min, termination of the reaction 4 °C – ∞. Expected lengths of the resulting fragments were 614 bp (for the long insert variant, the fragment A) and 604 bp (for the short insert variant, the fragment B) (Fig. 2a).

**Table 1:** List of primers used in this study

Number/name	Sequencing
YI-1/PVX pSg-INF_lg F	CCAGCTAGCAACAAACAAGAAAGGT**ATG**GCTACTCAACGAAGGG*
YI-2/PVX pSg-INF_sh F	ACCAGCTAGCA**ATG**GCTACTCAACGAAGGG*
YI-3/PVX NTR-INF R	ACGACCAAGCTCATTCCTTACTTCTTAAACTTTCT
YI-4/PVX INF-NTR F	GTAAGGAATGAGCTTGGTCGTATCACTGGAA
YI-5/PVX NTR-*Sac*I R	GCTGACAGAAGAGCTCACT**
-/INT FOR4	TTGATGCTCCTGGCACAG
-/INT REV2	TTCTGCTCTGACAACCTC

*Underline region is a unique restriction site for hydrolysis by the *Nhe*I restriction endonuclease, bold letters indicate a start codon of the *TP* sequence fused in ORF with the *inf α2b* gene

**Underline region is a unique restriction site for hydrolysis by the *Sac*I restriction endonuclease

The NTR fragment was prepared in the same manner. The fragment was amplified with a pair of primers YI-4 (forward) and YI-5 (reverse) (Table 1) using the pICH27566 plasmid vector as a template. Primers were design in such manner that the resulting PCR product had the NTR fragment with *Sac*I restriction site at the 3′-end and the 5′-overhang (an end fragment of the *inf α2b* gene). Other components of the PCR reaction mixture were the same as described supra for the *inf α2b* gene preparation. The PCR amplification program was as follows: initial denaturation 98 °C – 30 s, 35 cycles (denaturation 98 °C – 10 s, primer annealing 55 °C – 15 s, extension 72 °C – 5 s), final extension 72 °C – 7 min, termination of the reaction 4 °C – ∞. The expected length of the fragment C was 189 bp (Fig. 2b).

Then the overlapping PCR was performed to combine the fragment A/B (carrying the *TP-inf α2b* gene with the *Nhe*I restriction site) and the fragment C (the NTR sequence with the *Sac*I restriction site) in one insertion fragment for cloning in the plasmid acceptor vector. For that 1 µL of the amplified fragment A/B and 1 µL of the amplified fragment C (without any additional purification), 1x  Phusion HF Buffer, dNTP mix (200 µM each), pair of primers YI-1/YI-2 (forward) and YI-5 (reverse) (0.4 µM each), Phusion HF DNA Polymerase (0.4 U) and milliQ water to the final volume (to 50 µL) were mixed in one tube. The PCR amplification program was as follows: initial denaturation 98 °C – 30 s, 35 cycles (denaturation 98 °C – 10 s, primer annealing 55 °C – 20 s, extension 72 °C – 15 s), final extension 72 °C – 7 min, termination of the reaction 4 °C – ∞. Expected lengths of the resulting fragments D and F were 782 bp and 772 bp, correspondently (Fig. 2c). Then the obtained PCR fragments D and F (the prepared insertions) were purified using a commercial purification kit (NucleoSpin Gel and PCR Clean-up, Cat. No. 740609.50, Macherey–Nagel, Germany) and used for further work.

### The pPVX-INF plasmid vectors assembly and cloning in bacterial cells

Both the pICH27566 plasmid acceptor vector and the purified insertions (fragments D and F) were hydrolytically digested through the unique digestion sites by the restriction endonucleases *Nhe*I-HF and *Sac*I-HF (R3131S and R3156S New England Biolabs, UK). One restriction mixture contained 1x  Smart Buffer, *Nhe*I-HF (24 U), *Sac*I-HF (24 U), the purified plasmid acceptor vector or the purified insertion (16 µL), mQ (to 50 µL). To prevent evaporation, restriction (double digestion) was carried out in a PCR machine: 37 °C – 15 min, followed by 80 °C – 20 min to stop the reaction. The pre-treated insertions were ligated with the pre-treated plasmid acceptor vector: 1x  T4 DNA ligase buffer, the vector and the insertion (at 1:1.1 ratio), T4 DNA ligase (Cat. No. EL0011, Thermo Fisher Scientific). Incubated at room temperature for 1 h. The resulting PVX-based plasmid vectors carrying the *TP-inf α2b* gene (Fig. 1d), named as the pPVX-INF_long and pPVX-INF_short (with 5′UTR between the promoter and start codon, and without 5′UTR, correspondingly), were inserted in the bacterial competent cells of *Escherichia coli* strain XL1Blue (from the collection of the Institute of Cell Biology and Genetic Engineering, Kyiv, Ukraine) by heat-shock method (Froger and Hall 2007) for plasmid multiplication. Several *E. coli* colonies appeared on the plates with agar LB medium containing selective agent kanamycin (100 mg/L) after overnight incubation at 37 °C (Fig. 2d).

Presence of the resulting pPVX-INF plasmid vectors in the appeared *E. coli* colonies was confirmed by common PCR analysis with DreamTaq DNA Polymerase (Thermo Fisher Scientific) using the *inf α2b* gene-specific pair of primers INT FOR4 (forward) and INT REV2 (reverse) (Table 1). The PCR amplification program was as follows: initial denaturation 95 °C – 5 min, 35 cycles (denaturation 95 °C – 20 s, primer annealing 52 °C – 25 s, extension 72 °C – 25 s), final extension 72 °C – 1 min, termination of the reaction 4 °C – ∞. Expected length of the fragment was 396 bp (Fig. 2e). Additionally, we used a pair of primers INT FOR4 (forward) and YI-5 (reverse) to verify that the *inf α2b* gene was a part of the PVX-based plasmid vectors. The PCR amplification program was as follows: initial denaturation 95 °C – 5 min, 35 cycles (denaturation 95 °C – 20 s, primer annealing 55 °C – 25 s, extension 72 °C – 35 s), final extension 72 °C – 1 min, termination of the reaction 4 °C – ∞. The expected length of the fragment was 634 bp (Fig. 2f). Positive *E. coli* colonies were multiplied, and the pPVX-INF plasmids were isolated using a commercial purification kit (NucleoSpin Plasmid, Mini kit for plasmid DNA, Cat. No. 740588.50, Macherey–Nagel). The purified pPVX-INF plasmids were transferred in the competent cells of *Agrobacterium tumefaciens* strain GV3101 (from the collection of the Institute of Cell Biology and Genetic Engineering, Kyiv, Ukraine) by heat-shock method (Höfgen and Willmitzer 1988). Presence of the resulting pPVX-INF plasmid vectors in the appeared *A. tumefaciens* colonies was confirmed by PCR analysis as described supra for *E. coli* colonies. Positive colonies were multiplied and further used for plant infection.

### Production of hINF alpha 2b through *Agrobacterium*-mediated transient gene expression

To verify the functional activity of the created plasmid vectors and to check the level of accumulation of the recombinant hINF alpha 2b, the *Agrobacterium*-mediated transient expression method was used. *Nicotiana benthamiana* plants were infected with bacterial suspension carrying the created pPVX-INF plasmid vectors. As a positive control, we used *N. benthamiana* plants infected with bacterial suspension carrying the pICH27566 plasmid vector and accumulated the recombinant GFP. As the pICH27566 and the pPVX-INF plasmid vectors have the same backbone, we assumed that both recombinant proteins (GFP and hINF) would be produced at the same accumulation rate. GFP accumulation in the attached leaves was monitored under non-destructive conditions using UV light. We estimated that the highest level of GFP production was observed 7 days after inoculation, that is why we collected plant material with hINF at the same time (the 7th day post infiltration) when GFP expression was the highest.

**Fig. 3:**
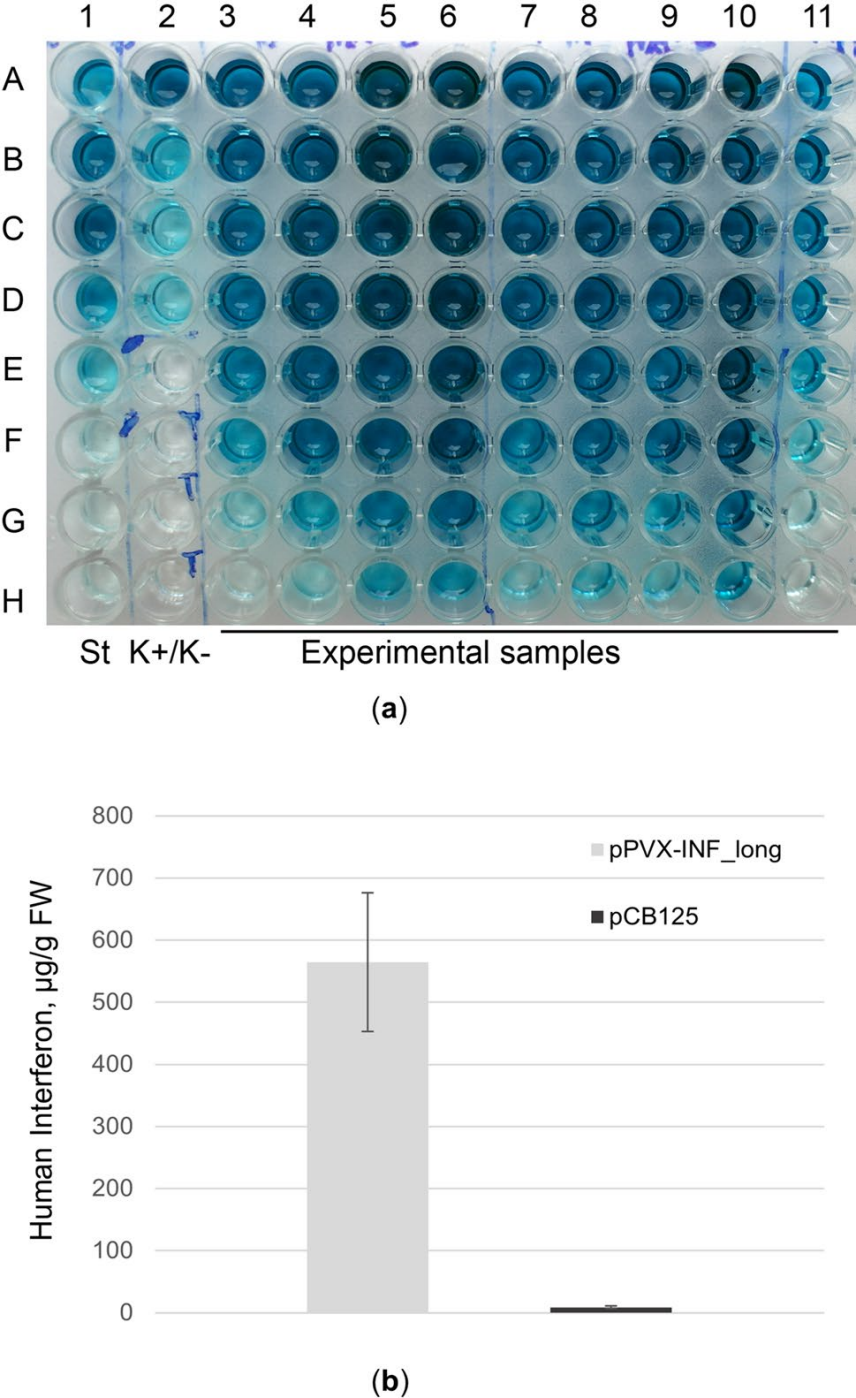
Quantitative assessment of recombinant hINF alpha 2b production in *N. benthamiana* plants. **a** Indirect ELISA: wells 1A-1H – a positive control: titration of standard (St) hINF alpha 2b in coating buffer, wells 2A-2D – a positive control: titration of standard hINF alpha 2b added to the total protein extract of wild type plant (K +), wells 2E-2H – a negative control: total protein extracts of wild type plant (K-), rows 3–10 wells A-H – twofold serial dilutions (1:20 – 1:2560) of experimental rude protein extracts prepared after infection of plants with pPVX-INF_long plasmid vector; wells 11A-11H – twofold serial dilutions (1:20–1:2560) of experimental rude protein extract prepared after infection of plant with pPVX-INF_short plasmid vector. The darker the color, the higher the level of hINF protein in the sample. **b** Comparison of hINF alpha 2b production using different expression cassettes: the created PVX-based expression system (pPVX-INF_long) and a simple expression system where the target gene was controlled by 35S promoter of CaMV (pCB125)

Using ELISA method and control dilutions of standard hINF, we evaluated the efficiency of the two created plasmid vectors (pPVX-INF_long and pPVX-INF_short) based on the accumulation of hINF alpha 2b in crude protein plant cell extracts. Based on the obtained results, we estimated that the production of hINF alpha 2b in *N. benthamiana* plants reached high levels with the PVX-based vector constructions. The average percentage of hINF alpha 2b to TSP was 5.98 ± 0.69% (the variation range was 3.64–9.38%) when the pPVX-INF_long vector was used, and 4.63 ± 0.59% TSP (the variation range was 3.13–5.95% TSP) when the pPVX-INF_short version was used. The mean of hINF alpha 2b per fresh leaf weight was 0.564 ± 0.112 mg/g FW (the variation range was 0.270–1.244 mg/g FW) (Fig. 3a) when the pPVX-INF_long vector was used, and 0.375 ± 0.038 mg/g FW (the variation range was 0.240–0.468 mg/g FW) when the pPVX-INF_short version was used. The highest value of hINF alpha 2b reached about 1.2 mg/g FW. There were no reliable differences between the hINF accumulation after using the two variants of PVX-based plasmid vectors, but the average mean was higher in case of using the pPVX-INF_long plasmid vector; therefore, we used this vector construction in the further work. We found that the PVX-based system is more effective for hINF alpha 2b production than the simple system where the *inf α2b* gene is controlled by strong constitutive 35S promoter of CaMV. According to our data the average percentage of hINF alpha 2b to TSP was 0.08 ± 0.02% (or 0.009 ± 0.002 mg/g FW) when the *inf α2b* gene was controlled by 35S promoter of CaMV (Fig. 3b). It means that hINF accumulation was about 80 times higher with the new created pPVX-INF_long plasmid vector comparing to the simple system controlled by 35S promoter of CaMV. The difference between the hINF production using different expression systems was highly reliable (*P* < 0.01). For an agroinfiltration procedure, we used the first tree fully expended leaves from the apex as these leaves were shown the best producers of recombinant proteins (Sindarovska and Kuchuk 2021). We estimated that the total yield of hINF that can be obtained from the three leaves of one plant is 0.671 ± 0.106 mg. We also confirmed by ELISA method that recombinant hINF accumulated in upper uninfected leaves due to systemic distribution of viral RNA carrying the *inf α2b* gene.

We also used the pPVX-INF_long plasmid vector for obtaining hINF alpha 2b in an edible plant species belonging to another taxonomic family, namely, *O. basilicum* (sweet basil). Recently, we demonstrated that sweet basil produces high levels of GFP when the pICH27566 plasmid vector is used (Sindarovska and Kuchuk 2023). We confirmed that the created pPVX-INF_long plasmid vector was functional in both plant species. hINF alpha 2b was produced in sweet basil plants as well, while the levels of expression were lower than in *N. benthamiana* plants and reached about 0.08 ± 0.02% TSP (the variation range was 0.02–0.15%) or 0.012 ± 0.003 mg/g FW (the variation range was 0.003–0.020 mg/g FW). The amount of hINF obtained in basil plants after using PVX-based expression vector system was similar to amount of hINF obtained in *N. benthamiana* after using the simple system where the *inf α2b* gene was controlled by 35S promoter of CaMV.

## Discussion

Although plants are considered promising hosts for the recombinant protein production, plant-made pharmaceuticals are still rare products on the global market as the levels of the produced valuable proteins are quite low. One of the factors that significantly affects the final yield of the recombinant protein is the efficiency of transgene expression. Successful expression of the target gene largely depends on the expression cassette used. It has been shown in many publications that plant expression vectors based on the genome elements of phytopathogenic viruses increase the final yield of the valuable target proteins (Jiang et al. 2019; Maharjan et al. 2021; Malla et al. 2021; Phakham et al. 2021; Yamada Y et al. 2020). Thus, we used the PVX-based expression vector that ensures high level of GFP accumulation (Sindarovska and Kuchuk 2021, 2023) as a backbone to create the pPVX-INF plasmid vectors. In our earlier works we used a different viral-based expression system to obtain recombinant hINF alpha 2b through *Agrobacterium*-mediated transient expression in other good host plant species *N. excelsior* and *N. cavicola* (Sindarovska et al. 2010, 2019). However, the levels of hINF obtained with an old expression system were about three orders lower compared to our new PVX-based expression system. Such results can be explained by a less efficient expression system rather than a host factor, as the created pPVX-INF_long plasmid vector works more efficiently in two plant species belonging to different taxonomic families.

As mentioned supra, many researchers used viral-based expression vectors to increase the levels of recombinant proteins. In this study, we confirmed that the expression system based on PVX genome elements works more efficiently for the production of recombinant hINF alpha 2b than a simple expression system where the gene of interest is controlled by constitutive 35S promoter of CaMV. The results accord with the data reported earlier: the final yield of recombinant proteins was higher when gene expression was controlled by multiple viral genome elements not only the strong 35S promoter of CaMV (Lindbo 2007a, b; Sindarovska et al. 2019; Sheludko et al. 2007). In this study, we compared production of the recombinant protein using two genetic vector constructions: with and without 5′UTR before the target gene. While there was no statistical difference between the obtained results, the average mean of hINF production was higher in case of the presence of 5′UTR in the vector construction. Our results agree with the results shown in other papers and confirm that 5′UTRs can increase recombinant protein production obtained through transient gene expression (Diamos et al. 2016; Jansing and Buyel 2019; Pfotenhauer et al. 2022). We also confirmed that hINF can produce in upper uninfected leaves; thus, we plan to use our constructions further for obtaining *N. benthamiana* plants expressing hINF in vitro culture (Sindarovska and Kuchuk 2021).

We compared the results about the production of interferons in plant systems obtained by other research groups with our own results. For example, the level of hINF alpha 2b produced by transgenic tobacco cells reached about 1.0–3.5% of TSP in cultural medium (Xu et al. 2007). While in chloroplasts of transgenic tobacco plants, the levels were reached up to 20% of TSP or about 3 mg/g FW (Arlen et al. 2007). Interferon production in our system was higher than in transgenic tobacco cells but lower than in transgenic tobacco chloroplasts. However, transgene expression in transgenic plants can stop at any time due to gene silencing, while transient gene expression is more reliable if viral suppressors of gene silencing are co-expressed with the gene of interest.

## Data Availability

The data supporting the findings of this study are available within the article.
